# A Reducing Substance in Urine Resembling Glucose

**Published:** 1887-01

**Authors:** T. Barton Brune

**Affiliations:** Baltimore, Md.


### *A REDUCING SUBSTANCE IN URINE RESEMBLING GLUCOSE.*

A few days ago a specimen of urine was sent to the writer, the analysis of which revealed the presence of a substance likely to be of importance to life insurance companies and of considerable interest to individuals applying for insurance. On the 13th of this month, the agent in this city, of one of the prominent New York life insurance companies sent me a sample of urine and the following account of it. It was passed by a young man of good physique, who had, in the last three years, gained fifteen pounds in weight and three inches in abdominal girth and also was in apparently perfect health, but had been repeatedly turned down, as a bad risk, by prominent medical examiners of other first-class companies, because of the presence of glucose in his urine. As he had recently been examined by the medical staff of the agent's company, and some doubt had been raised regarding the presence of glucose in his urine and as he wished a large insurance the agent requested me to analyze the accompanying sample and communicate to him the result of the analysis. The analysis, made December 13, 14 and 15, was as follows:

### ANALYSIS OF THE URINE.

*Physical characteristics.*—Urine sparklingly clear, light amber in color, urinous smell, acid reaction, specific gravity 1022; amount for twenty-four hours said to be 32 ounces. No sediment when received. Applicant on ordinary mixed diet, having eaten nothing unusual and taken no medicine.

*Normal Constituents.*—Indican and other normal ingredients of urine present in normal quantities, as determined approximately. No bile pigment as determined by sulphuric acid and nitric acid containing little nitrous acid. No albumen.

Upon standing several hours (fourteen) a very slight, floculent, whitish sediment appeared, and the supernatant fluid began to be discolored, a reddish yellow, later a brownish-red, from its surface down.

*Heller's or Moore's Test.*—Upon the addition of a solution of *sodic hydrate* (1-3) the earthy phosphates were precipitated in considerable quantity and the supernatant fluid became a port wine red, later (after a few hours) almost black. This effect was hastened by *boiling*. *Nitric acid* in small quantity rather heightened the color, but in excess almost decolorized it without evolving any caramel odor. The sodic hydrate was proven free from lead salts, and the urine free from hydrogen sulphide. Probably from this reaction a considerable quantity of grape sugar was supposed, by some examiners, to have been present.

*Boetger's Test.*—Upon the addition of *subnitrate* of *bismuth* and solution of *sodic hydrate* in the cold, a reaction took place in the supernatant fluid similar to that above, but the subnitrate was not abnormally discolored even upon *boiling*, becoming simply a very light brown.

*Robert's Modification of Fehling's Test.*—Upon boiling 4-5 c.c. of Fehling's Test Fluid, as recommended by Roberts, and adding 1-2 c.c. urine there first appeared a dark, greenish discoloration rapidly followed by separation of a greenish-white sediment from a dark brown supernatant fluid. On standing an hour its sediment resolved itself into a greenish-white layer superimposed upon a mixed red and orange layer, a reaction resembling that of sugar.

*Johnson's Picric Acid Test.*—Ten minims of *picric acid* solution gave, according to Johnston's method, a reaction similar to that produced by one and a half grains of glucose, and twenty minims a reaction such as would be caused by two grains per ounce of urine. In both cases, however, the discoloration ensued before as well as after boiling, and the resulting color was more brown and less red than that produced by glucose.

*Fehling's Test.*—With *Fehling's* test a very unsatisfactory reaction ensued, for while a slight amount of cuprous oxide was precipitated, instead of decoloration of the supernatant fluid there resulted a deep brown discoloration, similar to that produced by too long boiling of the fluid in Robert's test.

*Fermentation* gave no result, even after thirty-six hours.

*Ultzmann's Saccharimeter.*—The *polariscope* showed, upon repeated trials, a lœvo-rotation 0.2°, a rotation, although the opposite of that caused by glucose, too slight to be of value.

*Thymol* and *sulphuric acid* gave a light violet color.

Upon the addition of *silver nitrate*, a grayish-white precipitate resulted, which quickly blackened upon agitation.

*Ferric chloride* gave a faint green precipitation.

*Nitric acid* gave a light yellow instead of violet purple zone between its fluids.

*Boiling* gave a reddish purple, clear fluid.

*Galippe's Test.*—*Picric acid* gave the faintest possible white-cloudiness, which cleared upon boiling, and no abnormal darkening of the mixture.

The *microscope* revealed that the sediment consisted of mucus with urates and some irregular crystals, and a few pigmented granules.

CONCLUSION.

The presence of glucose indicated (shall I say to the non-specialists?) by the sodic-hydrate, copper, and, perhaps, picric acid, and bismuth-subnitrate tests, is absolutely negatived by the polariscope, the fermentation, silver-nitrate and ferric-chloride tests, and a proper interpretation of the other tests. Clearly, the question as to the presence of glucose is answered in the negative. As to the next question. What is the abnormal substance giving these reactions? I cannot answer without further analysis, but hope to supplement this by an account of its discovery. Whatever it is, it would seem to be in small quantity, and to have little, if any, pathological significance. The brownish discoloration of the urine from above downwards, and its reaction with sodic hydrate and its copper salts, would indicate a substance discovered in urine by Baedeker, in 1861, and called "alkapton." This body is, however, probably a composite one, and its further reaction with ferric chloride and silver nitrate would point towards pyrocatechol or proto-cateclinic acid, (both of exceedingly rare occurrence in its human urine) as the possible cause of the behavior of the urine. The analysis, however, is not complete. Although such is the case, two important lessons may be drawn from this analysis:—The one is that the copper and sodic hydrate tests, as generally used, are not sufficient *positive* tests for glucose, but should be supplemented by the fermentation, subnitrate of bismuth, and polariscope tests, or, in doubtful cases, by all three; the other is that medical examiners should not lightly tell applicants for insurance that they are suffering from serious illness. For this applicant was very much alarmed by a very prominent examiner in another city telling him he was in the last stages of diabetes and absolutely refusing to recommend him for insurance.—T. Barton Brune, M. D., Baltimore, Md., in *Boston Medical and Surgical Journal.*

